# NEW TOOL ENHANCES DETECTION OF LIFE-EXTENDING INTERVENTIONS BY DELINEATING THEIR TEMPORAL EFFICACY

**DOI:** 10.1093/geroni/igae098.2007

**Published:** 2024-12-31

**Authors:** Nisi Jiang, Qianqian Liu, Catherine Cheng, Randy Strong, Jonathan Gelfond, James Nelson

**Affiliations:** UT Health San Antonio, San Antonio, Texas, United States; UT Health San Antonio, San Antonio, Texas, United States; UT Health San Antonio, San Antonio, Texas, United States; UT Health San Antonio, San Antonio, Texas, United States; UT Health San Antonio, San Antonio, Texas, United States; University of Texas Health at San Antonio, San Antonio, Texas, United States

## Abstract

Drug discovery of anti-aging interventions relies on survival data, which is time-consuming and costly to generate, yet the analysis of such data often fails to capitalize on its full potential. The predominant method for assessing intervention effects on lifespan, the log-rank test, lacks the power to identify when and for how long an intervention is effective. Moreover, it has decreased sensitivity for interventions that are only effective during part of the life course. Statistical tools are needed to address these limitations. They should be capable of identifying when, for how long, and to what extent an intervention reduces (or increases) mortality risk. Here we introduce a Temporal Efficacy Profiler (TEP), which meets these needs, and apply it to survival data from 42 compounds tested in the NIA Interventions Testing Program (ITP) using UM-HET3 mice. Compared to the log-rank test, this tool identified over twice as many compounds that increased (or decreased) survival, largely because of its sensitivity to variable efficacy of the interventions across the life course. Sex differences were prevalent. The TEP revealed agents that either reduced (22 compounds) or increased (15 compounds) mortality hazards, with some (2 compounds) showing dual effects depending on sex. Notably, the efficacy of these interventions varied significantly in both duration and magnitude. Moreover, the TEP uncovered adverse effects that were missed by the log-rank test. Of note, only 8 compounds significantly reduced mortality hazards beyond the 90th percentile of mortality, a period when the burden of senescence is highest.

